# The DPY-14 cuticle collagen regulates left-right asymmetric neuronal migration in *Caenorhabditis elegans*

**DOI:** 10.17912/micropub.biology.001302

**Published:** 2024-08-26

**Authors:** Erik Lundquist

**Affiliations:** 1 Molecular Biosciences, University of Kansas, Lawrence, Kansas, United States

## Abstract

Nervous systems of bilaterally-symmetric animals display left-right asymmetries in development. In *Caenorhabditis elegans* , the Q neuroblasts display left-right asymmetry of migration, with QR on the right migrating anteriorly and QL on the left migrating posteriorly. Previous worked showed that a group of transmembrane receptor molecules including UNC-40 /DCC and PTP-3 /LAR control direction of initial Q migration. However, no classical secreted paracrine growth factor has been identified. Previous work showed that molecules in the extracellular matrix are involved, including UNC-52 /Perlecan and the cuticle collagens DPY-17 and SQT-3 . This report shows that the cuticle collagen DPY-14 is also involved, and genetically acts with DPY-17 and SQT-3 , possibly in a collagen trimer. DPY-14 might be a component of an inherent left-right chirality in the extracellular matrix that directs left-right asymmetric Q neuroblast migration.

**Figure 1. AQR and PQR migration.**
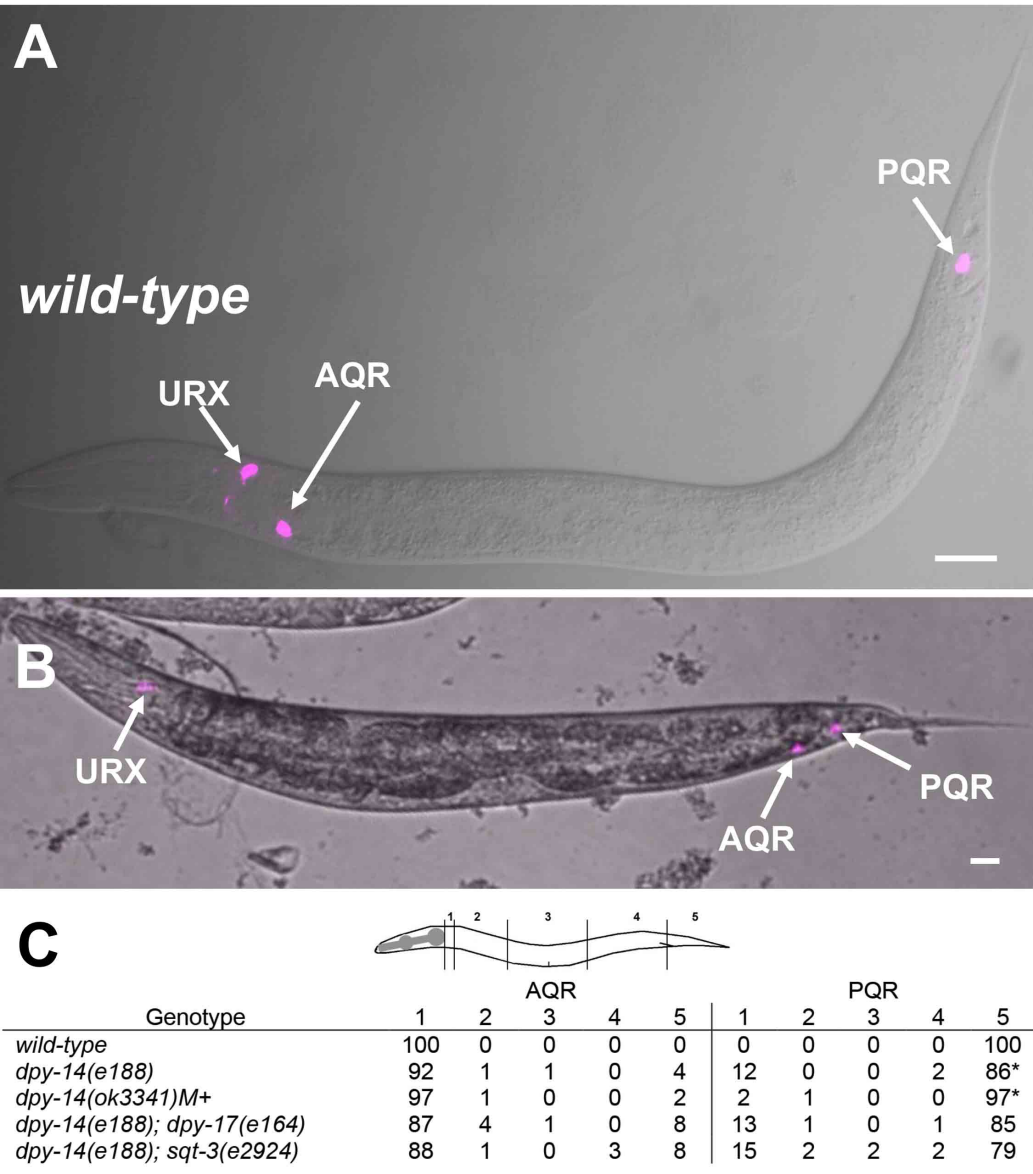
*
The lqIs244 [Pgcy-32::cfp]* transgene was used to visualize AQR in the head and PQR in the tail. A) A wild type larva showing AQR position and PQR position. The URX neurons also express *Pgcy-32::cfp* . B) A *dpy-14 ( e188 )* larva with AQR that has migrated posteriorly toward the tail. The scale bars represents 10μM. C) Quantification of AQR and PQR migration in different genotypes. For each genotype, 100 animals were scored. The locations of AQR and PQR along the body were scored on a 5-position scale (see diagram). 1 is the wild-type position of AQR; 2 is posterior to the normal AQR position but anterior to the vulva; 3 is at or near the vulva; 4 is the normal birth position of the Q neuroblasts; and 5 is the wild-type position of PQR. In pairwise comparisons using Fisher's exact test, there were no significant differences at positions 1 and 5 between *dpy-14 ( e188 )* and double mutants with *dpy-17 ( e164 )* or *sqt-3 ( e2924 ).* *dpy-14 ( ok3341 )M+* was significantly different from *dpy-14 ( e188 )* at PQR position 5 ( *p* = 0.01) (asterisk).

## Description

The bilateral Q neuroblasts are sisters of the V5 seam cells and are born during embryogenesis (Sulston and Horvitz 1977; Chapman *et al.* 2008; Middelkoop and Korswagen 2014). At larval hatching, QR on the right protrudes and migrates anteriorly over the V4 seam cell, and QL on the left protrudes and migrates posteriorly over the V5 seam cell (Chapman *et al.* 2008). QR and QL then undergo an identical pattern of cell division, cell death, and neuronal differentiation to produce three neurons each. QR produces AQR, AVM, and SDQR, and QL produces PQR, PVM, and SDQL. QR daughters migrate anteriorly, including AQR which migrates the longest distance to the deirid ganglion near the pharynx ( Figure 1A ). QL daughters migrate posteriorly, with PQR migrating the longest distance to the phasmid ganglion posterior to the anus ( Figure 1A ).

A group of transmembrane receptor molecules act together to control the initial migration of QR and QL, including UNC-40 /DCC, PTP-3 /LAR, MIG-21 , and the Cadherins CDH-3 and CDH-4 (Middelkoop *et al.* 2012; Sundararajan and Lundquist 2012; Sundararajan *et al.* 2014; Ebbing *et al.* 2019). In QL, UNC-40 /DCC and PTP-3 /LAR act redundantly in parallel to drive posterior protrusion and migration. In QR, UNC-40 and PTP-3 mutually inhibit one another's posterior migration activity, resulting in anterior protrusion and migration. Defects in the direction of initial migration affects the subsequent migration of AQR and PQR (Chapman *et al.* 2008). *unc-40 , ptp-3 , mig-21 ,* and *cdh-4* mutants each have misdirected AQR and PQR migration, with AQR sometimes migrating posteriorly and PQR sometimes migrating anteriorly.

Previous studies indicate that the cuticle collagen genes *dpy-17* and *sqt-3* result in initial QL and QR migration defects similar to *unc-40 , ptp-3 , mig-21 ,* and *cdh-4* (Lang and Lundquist 2021) . *dpy-17* and *sqt-3* encode similar single collagen repeat molecules of the collagen IV family (Novelli *et al.* 2006; Fotopoulos *et al.* 2015). Work described here shows that the *dpy-14* is also required AQR and PQR migration similar to *dpy-17* and *sqt-3 .* *dpy-14 ( e188 )* has a morphological phenotype similar to *dpy-17* and *sqt-3* : a spindle-shaped Dpy that is very severe in early larval development and that gets less severe as the animals develop to adulthood (Gallo *et al.* 2006). *dpy-14* encodes a cuticle collagen with a single collagen repeat similar to DPY-17 and SQT-3 (Gallo *et al.* 2006). *dpy-14 ( e188 )* mutants displayed defects in AQR and PQR migration, with reversals of direction of migration of both cells: 6% of AQRs migrated posteriorly to the normal position of PQR ( Figure 1B and C); and 11% of PQR migrated anteriorly to the normal position of AQR ( Figure 1C ). This is similar to the level of defects observed in *dpy-17* and *sqt-3* mutants (Lang and Lundquist 2021) . A deletion of the *dpy-14* gene, *dpy-14 ( ok3341 )* , resulted in sterile Dpy adults. *dpy-14 ( ok3341 )* animals with wild-type maternal *dpy-14* activity also displayed AQR and PQR migration reversals, with AQR migration significantly less severe than *dpy-14 ( e188 )* ( Figure 1C ). This difference could be due to wild-type maternal *dpy-14* activity in *dpy-14 ( ok3341 ).* However, *dpy-14 ( e188 )* is a missense mutation that changes glycine 139 to arginine in the first collagen Gly–X–Y region (Gallo *et al.* 2006). Gly–X–Y repeats are required for trimerization of collagen monomers to form the collagen triple-helix (Bella *et al.* 1994; Bella *et al.* 2006). These data suggest that *dpy-14 ( e188 )* might not be a simple loss of function of *dpy-14* and might have some dominant interfering activity, possibly with *dpy-17* and/or *sqt-3 .*

Double mutants of *dpy-17* and *sqt-3* were no more severe than single mutants alone (Lang and Lundquist 2021) . *sqt-3 ; dpy-14 ( e188 )* and *dpy-17 ; dpy-14 ( e188 )* double mutants were also no more severe than either single alone (Table 1). The triple mutant could not be constructed and is likely inviable. In any event, these results suggest that DPY-14 , DPY-17 , and SQT-3 all act together to regulate AQR and PQR migration. DPY-17 and SQT-3 are thought to act together in a trimer (Novelli *et al.* 2006). One attractive hypothesis is that each of these three Collagen monomers form a Collagen triple helix molecule that acts in AQR and PQR migration.

No classical secreted paracrine factor has been identified that regulates the left-right asymmetry of Q neuroblast migration, although the basement membrane heparan sulfate proteoglycan UNC-52 /Perlecan is involved (Ochs *et al.* 2022), and this work and previous work (Lang and Lundquist 2021) suggests that the collagen extracellular matrix is involved. Possibly, the Q neuroblasts are responding to inherent left-right chirality that is present in extracellular matrices including the cuticle (Bergmann *et al.* 1998) to migrate anteriorly on the right and posteriorly on the left. UNC-52 /Perlecan and a DPY-17 / SQT-3 / DPY-14 collagen trimer might be part of this extracellular matrix left-right chirality to which the Q neuroblasts respond.

## Methods

Standard *C. elegans* genetics and culture techniques at 20°C were utilized (Brenner 1974) . AQR and PQR were visualized using *Pgcy-32::cfp* transgenes (Chapman *et al.* 2008; Josephson *et al.* 2016). AQR and PQR position along the body was noted using a five-position scale as previously described (Josephson *et al.* 2016; Lang and Lundquist 2021) (see Figure 1C ): position 1 is the normal position of AQR in the deirid ganglion in the anterior; position 2 is posterior to the normal position of AQR but anterior to the vulva; position three is around the vulva; position four if the birthplace of the Q neuroblasts in the posterior; and position 5 is the normal final position of PQR in the tail in the phasmid ganglion behind the anus.

## Reagents

The following *C. elegans* mutants and variants were used: LGI, *dpy-14 ( e188 ), dpy-14 ( ok3341 ), tmC20 ;* LGII, *lqIs244 [Pgcy-32::cfp];* LGIII, *dpy-17 ( e164 );* LGV, *sqt-3 ( e2924 )* . The *tmC20* balancer (Dejima et al., 2018) was used to maintain *dpy-14 ( ok3341 )* heterozygotes *.* The following strains were analyzed:

**Table:** 

Strain	Genotype	Origin
N2	*wild-type*	CGC
LE6871	*dpy-14 ( e188 ) I; lqIs244 II *	CGC/this work
LE6873	*dpy-14 ( ok3341 )/ tmC20 I; lqIs244 II *	CGC/this work
LE7089	*dpy-14 ( e188 ) I; sqt-3 ( e2924 ) V; lqIs244 II *	CGC/this work
LE6879	*dpy-14 ( e188 ) I; dpy-17 ( e164 ) III; lqIs244 II *	CGC/this work
